# Crack densification in drying colloidal suspensions

**DOI:** 10.1126/sciadv.adp3746

**Published:** 2024-09-11

**Authors:** Paul Lilin, Mario Ibrahim, Irmgard Bischofberger

**Affiliations:** Department of Mechanical Engineering, Massachusetts Institute of Technology, Cambridge, MA 02139, USA.

## Abstract

As sessile drops of aqueous colloidal suspensions dry, a close-packed particle deposit forms that grows from the edge of the drop toward the center. To compensate for evaporation over the solid’s surface, water flows radially through the deposit, generating a negative pore pressure in the deposit associated with tensile drying stresses that induce the formation of cracks. As these stresses increase during drying, existing cracks propagate and additional cracks form, until the crack density eventually saturates. We rationalize the dynamics of crack propagation and crack densification with a local energy balance between the elastic energy released by the crack, the energetic cost of fracture, and the elastic energy released by previously formed cracks. We show that the final spacing between radial cracks is proportional to the local thickness of the deposit, while the aspect ratio of the crack segments depends on the shape of the deposit.

## Teaser

An energy criterion governs crack densification in drying drops of a colloidal suspension, resulting in complex crack patterns.

## INTRODUCTION

The beauty of desiccating cracks can be appreciated in mud fields, cemented floors, and peeling paint on old walls (*1*–*8*). The evaporation of the solvent from a film of a colloidal suspension induces the buildup of a solid deposit of close-packed particles (*9*–*11*). This deposit fractures into a diversity of patterns depending on the properties of the suspension and of the substrate and on the shape of the deposit (*12*–*21*). Controlling the morphology and spacing of the cracks, or conversely measuring the obtained crack pattern to infer properties of the suspension, has applications in microfabrication, medical diagnostics, and art conservation (*3*, *22*–*24*). The crack pattern forms incrementally, with successive generations of cracks dividing the deposit into smaller fragments (*25*–*29*). Revealing the mechanisms that govern the step-by-step formation of the cracks is key to a fundamental understanding of the final pattern.

The contact line of a drop of a concentrated colloidal suspension on a hydrophilic substrate remains pinned to the substrate (*30*). A capillary-driven flow brings particles to the edge of the drop, where they assemble into a close-packed deposit that grows radially inward and eventually covers almost the entire initial drop area (*10*, *15*, *20*, *31*). As the deposit grows inward, it remains saturated with water, and water lost to evaporation at the top of the deposit is replaced by water from the liquid region (*32*, *33*). The flow of water is sustained by a lower pressure inside the deposit compared to that in the liquid region (*34*–*36*). This liquid pressure in the pores of the deposit causes the deposit to shrink vertically, while horizontal shrinkage is prevented by adhesion to the substrate, which leads to in-plane tensile stresses that increase as the deposit grows (*6*, *11*, *37*–*40*). A first crack forms in the deposit when the elastic energy released by the crack becomes larger than the energetical cost of fracturing (*36*). The drying stresses increase with continued evaporation, and additional cracks form, creating an intricate final crack pattern (*15*, *36*, *41*).

The final crack pattern is characterized by the spacing and the orientation of the cracks. The crack spacing observed in dried deposits generally increases linearly with the deposit thickness (*3*, *18*, *20*, *42*–*45*). These experiments though were conducted either with a uniform deposit thickness or by measuring the thickness and crack spacing at a single location in a dried drop, and it is unknown how the crack spacing adapts locally to a varying deposit thickness. Variations in the thickness can cause cracks to branch, arrest, or oscillate (*16*, *20*, *46*). The orientation of the cracks is typically perpendicular to the solidification front but can vary depending on the elastic properties of the substrate and the deposit and because of spatial variations in deposit thickness (*19*, *47*–*49*). The final crack pattern is the result of sequential crack formation, a process that has been studied for uniform deposits under a uniformly increasing drying stress but not for drying drops where the drying stress varies radially along the deposit (*27*, *29*). Because a crack locally relieves the stresses, creating additional cracks requires a progressively larger drying stress (*25*). Models predict that the ratio between the deposit thickness and the crack spacing increases with drying stress and eventually reaches a constant value, a phenomenon called crack saturation that explains the linear dependence of crack spacing on deposit thickness frequently observed (*27*, *50*–*54*). The transition to this saturated regime, however, has not been observed for a drying colloidal suspension.

Here, we combine precision measurements of the crack pattern and of the deposit thickness profile to show that the deposit thickness locally controls the final crack spacing, while the deposit shape controls the crack orientation. We show that the propagation and densification of cracks in drying drops can be rationalized by considering the drying stress induced by the pore pressure in the deposit, which we calculate using a hydrodynamic model that accounts for the viscous pressure loss due to flow inside the deposit. Cracks that form perpendicular to the direction of water flow in the deposit obstruct the flow and change the pore pressure, creating a two-way coupling between crack formation and drying stresses. We show that the ratio between the elastic energy available for fracture and the energetic cost of fracture governs the increase and saturation of the ratio of the deposit thickness and crack spacing in both the dynamic evolution of the crack spacing and the final pattern.

## RESULTS

### Final crack pattern governed by deposit shape

**Fig. 1.**
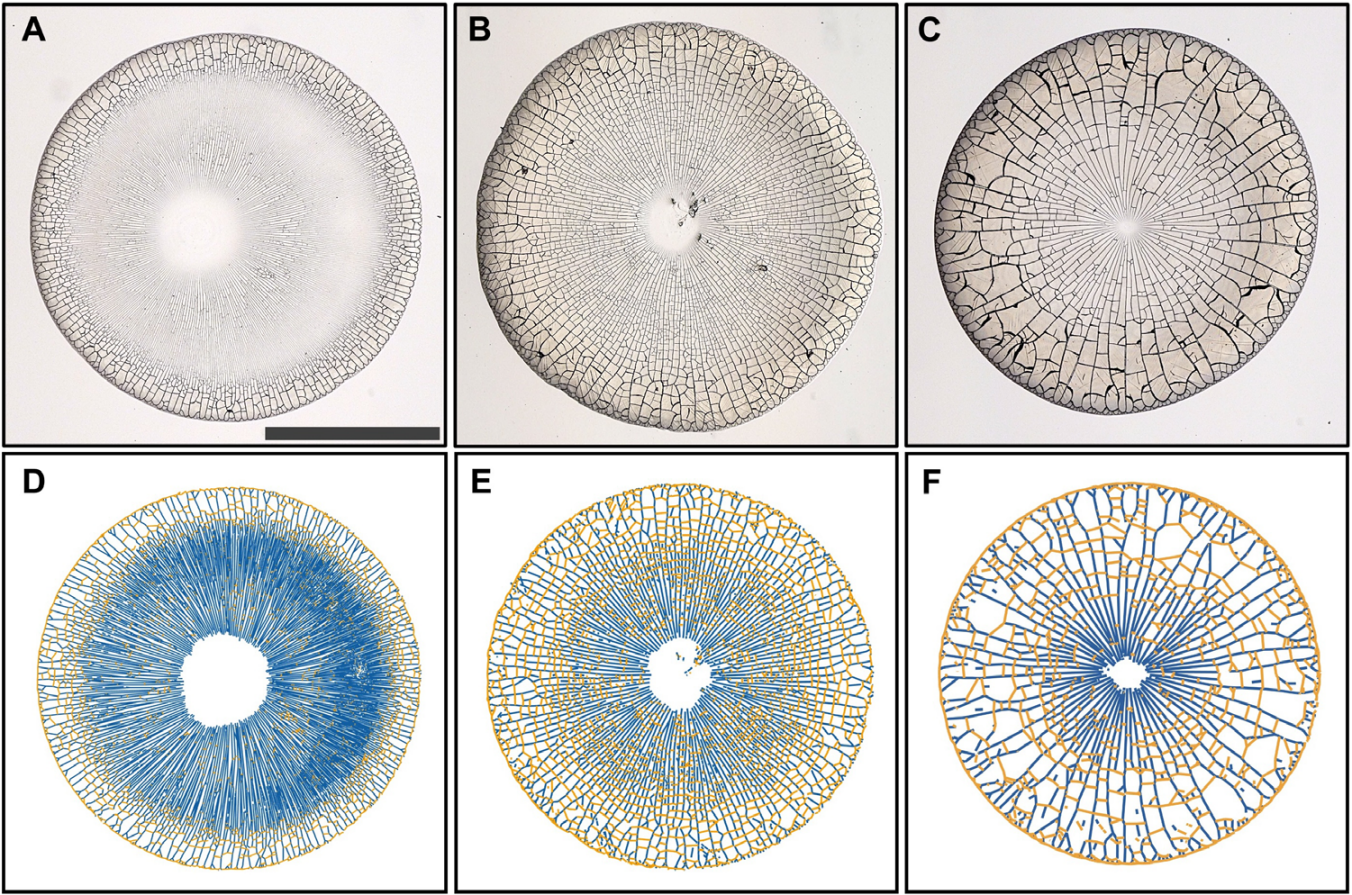
Dried deposits of drops of aqueous polystyrene particle suspensions. (**A** to **C**) Bottom view of dried deposits for drops with initial volume 0.3 μl and initial particle volume fractions ϕ_0_ = 0.03 (A), ϕ_0_ = 0.07 (B), and ϕ_0_ = 0.10 (C). Cracks form in the radial and orthoradial directions relative to the center of drying. The spacing between cracks increases with ϕ_0_. The scale bar denotes 1 mm. (**D** to **F**) Analyzed crack patterns scaled into a circle of radius unity centered on the center of drying. Radial cracks are highlighted in blue; orthoradial cracks are highlighted in orange.

To explore the range of crack pattern morphologies formed by drying drops of colloidal suspensions, we place drops of an aqueous suspension of polystyrene particles on a glass substrate. As water evaporates from the drop, the contact line of the drop remains pinned, and the particles assemble into a solid deposit of radius *r*_0_ = 1000 ± 80 μm. Cracks form in this deposit, creating a complex pattern that covers almost the entire initially wetted area, as shown in movie S1. For increasing initial particle volume fraction ϕ_0_ between 0.03 and 0.10, the spacing between the cracks increases and the area devoid of cracks in the center of the drop decreases, as shown in Fig. 1 (A to C). The orientation of the cracks is set by the circular drop geometry: Cracks orient preferentially along either the radial or the orthoradial direction with regard to a point in the center of the drop that we refer to as the center of drying. To facilitate the comparison of crack patterns from different drops, we scale the crack pattern by *r*_0_ such that it fits into a circle of radius unity centered on the center of drying. Radial cracks, shown in blue, are more closely spaced toward the center of the deposit than at the edge, as shown in Fig. 1 (D to F) and fig. S1. By contrast, orthoradial cracks, shown in orange, form predominantly farther away from the center of the deposit.

**Fig. 2.**
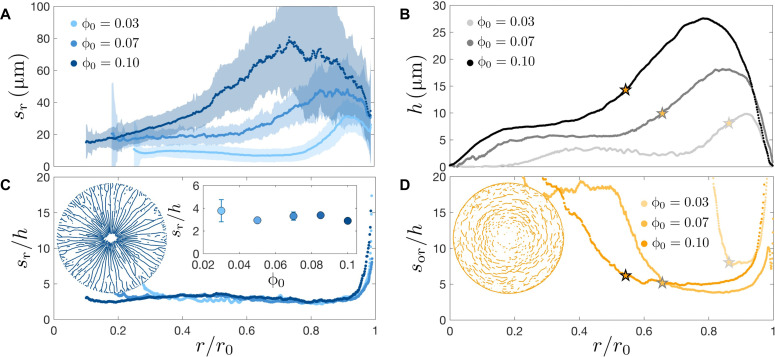
Dependence of crack spacing on deposit thickness. (**A**) Radial crack spacing *s*_r_ versus the normalized distance from the center of the deposit *r*/*r*_0_, where *r*_0_ is the deposit radius. The shaded region indicates the SD of the radial crack spacing distribution, as detailed in fig. S2. No cracks form in the center of the drop. (**B**) Deposit thickness *h* versus *r*/*r*_0_. The *r*/*r*_0_ dependence of the deposit thickness mirrors that of the radial crack spacing. The star symbols correspond to the *r*/*r*_0_ values defined in (D). (**C**) Ratio of the radial crack spacing and deposit thickness *s*_r_/*h* versus *r*/*r*_0_. *s*_r_/*h* is constant and equal to 3.1 ± 0.7 for most of the deposit. Inset: Average value of *s*_r_/*h* measured in the region of constant *s*_r_/*h* for multiple experiments at varying initial particle volume fractions ϕ_0_. The error bars indicate the SD. (**D**) Ratio of the orthoradial crack spacing and deposit thickness *s*_or_/*h*. *s*_or_/*h* is constant in a region toward the edge of the deposit that corresponds to the bump in the deposit thickness (indicated by a star) and increases toward the center of the deposit.

We begin to quantify the crack pattern by measuring the polar angle averaged spacing between radial cracks *s*_r_ at each distance *r* from the center of the drop, as detailed in fig. S2. The radial crack spacing *s*_r_ increases with initial particle volume fraction ϕ_0_ and exhibits a bump toward the edge of the drop, as shown in Fig. 2A. The SD of the spacing between two adjacent radial cracks is large as the crack spacing varies through the formation of additional cracks in between preexisting cracks, which halves the local crack spacing.

To understand the variation in crack spacing with ϕ_0_ and *r*, we measure the thickness of the dried deposit using an optical profilometer. Notably, the deposit thickness *h* mirrors the profile of the radial crack spacing *s*_r_, as shown in Fig. 2B, with a bump at large *r*/*r*_0_ and an increase in the deposit thickness *h* with increasing particle volume fraction. The radial crack spacing is proportional to the deposit thickness; we find a constant value *s*_r_/*h* = 3.1 ± 0.7 for most of *r*/*r*_0_ that is independent of the initial particle volume fraction, as shown in Fig. 2C. This result extends previous findings in uniform deposits on stiff substrates, where the crack spacing was reported to be proportional to the deposit thickness with a proportionality constant between 2 and 10 (*3*, *18*, *20*, *42*, *43*, *45*). It is remarkable that this proportionality holds locally despite the large variations in deposit thickness along the radial direction. The proportionality is only broken in the region of decreasing deposit thickness at the outer edge of the deposit, likely because of the rapid change in deposit thickness occurring at the edge.

Compared to radial cracks, orthoradial cracks have a larger spacing *s*_or_, indicating that the fragments are more elongated in the radial direction. *s*_or_ is also locally proportional to the deposit thickness, but only in a limited region toward the edge of the drop, as shown in Fig. 2D. The region of the deposit where orthoradial cracks are prominent and *s*_or_/*h* is constant corresponds to the bump in the deposit profile. The onset of this region and the corresponding deposit thickness vary for experiments with different initial particle volume fractions, which indicates that it is the shape of the deposit that governs the transition from dense orthoradial cracks spaced proportionally to *h* to sparse orthoradial cracks.

### Crack dynamics governed by pore pressure

The drying of a drop is a directional process characterized by multiple fronts that radially propagate. Right after the drop is deposited on the substrate, enhanced water evaporation at the edge of the drop and pinning of the drop contact line induce the initial formation of a solid particle deposit around the edge of the drop, while the center of the drop remains liquid (*10*, *30*). The solid particle deposit expands inward as particles from the liquid region at the center of the drop accumulate into it (*34*–*36*, *55*). As the drop dries and water evaporates from the deposit, the lost volume must be replenished either by air or by water from the liquid region. At the surface of the deposit, microscopic water-air menisci are pinned between the particles (*56*). Surface tension initially prevents air from penetrating inside the deposit, and water flows from the liquid region in the center of the drop through the deposit to compensate for evaporation over the surface of the water-saturated deposit (*32*, *33*). The viscous dissipation induced by this radial flow of water inside the pores of the deposit is at the origin of the propagating solidification and crack fronts (*35*, *36*).

**Fig. 3.**
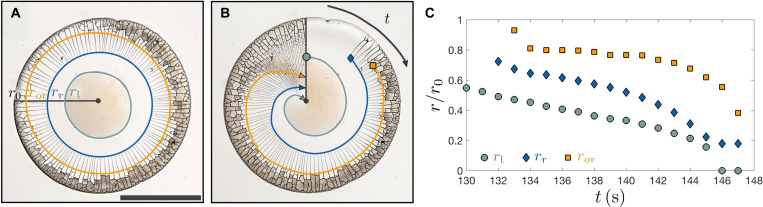
Dynamics of drying. (**A**) Bottom view of a ϕ_0_ = 0.05 drop at time *t* = 134 s since deposition. The deposit radius is *r*_0_. The solidification front that separates the central liquid region of radius *r*_l_ from the solid deposit is highlighted in gray. Radial cracks propagate at radius *r*_r_, defining the radial crack propagation front highlighted in blue. Orthoradial cracks form at a larger radius *r*_or_ highlighted in orange. Segments of deposit at radii *r* > *r*_or_ are opaque because of air replacing water in the deposit. (**B**) Time-lapse reconstruction image showing how the drying progresses from *t* = 130 s to *t* = 148 s. The radii of the liquid region *r*_l_ (gray), radial crack propagation front *r*_r_ (blue) and orthoradial crack propagation front *r*_or_ (orange) at the time *t* corresponding to the image section are indicated. (**C**) Time evolution of the liquid region with radius *r*_l_, radial crack propagation front *r*_r_ and orthoradial crack propagation front *r*_or_. Several events mark the drying dynamics: Radial cracks first form at *t* = 132 s, orthoradial cracks first form at *t* = 133 s, and the liquid region disappears at *t* = 146 s after drop deposition.

As the liquid region of radius *r*_l_ continues to shrink, cracks form in the solid particle deposit. The radial and orthoradial cracks differ in their dynamics. Radial cracks form first and propagate inward up to a distance *r*_r_ from the center of the drop. Orthoradial cracks form between the radial cracks at a later time, forming a front of radius *r*_or_ that separates the region of the deposit with and without orthoradial cracks. The three radii *r*_l_, *r*_r_, and *r*_or_ are indicated on the snapshot of the drying process shown in Fig. 3A. The entire drying process is shown in Fig. 3B by using a time-lapse reconstruction technique (*41*), where adjacent sectors of consecutive images are combined to create the composite image highlighting the propagation of the solidification, radial crack, and orthoradial crack fronts. We use our crack identification analysis on difference images to extract the location and orientation of new cracks at each point in time (see details in section S3 of the Supplementary Materials), as shown in Fig. 3 (B and C). The radial crack propagation front *r*_r_ is defined as the location where *s*_r_/*h* = 20, and the orthoradial crack propagation front *r*_or_ is defined as the location where *s*_or_/*h* = 20. The liquid region shrinks faster toward the end of drying, and the radial cracks propagate closer to the liquid region over time. We will show how these characteristics can be rationalized by considering the flow of water inside the porous deposit.

**Fig. 4.**
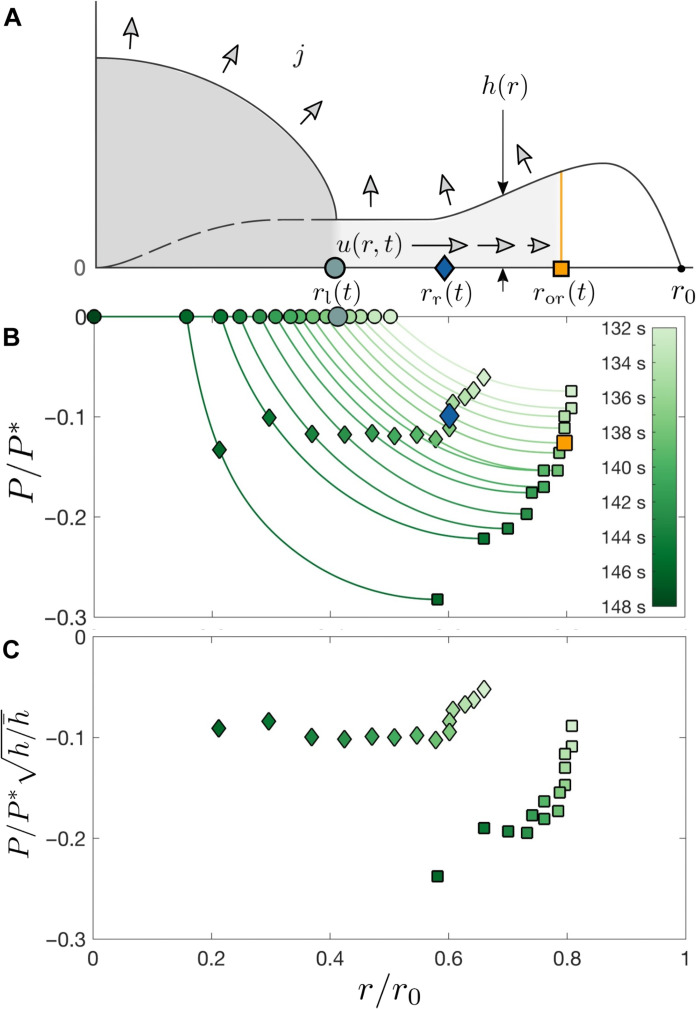
Drying-induced pore pressure and crack fronts. (**A**) Cross-sectional schematic of the drying drop. *u*(*r*, *t*) denotes the radial flow velocity through the porous deposit, *j* is the evaporative flux, and *h*(*r*) is the deposit thickness. *r*_l_(*t*) denotes the radius of the solidification front that separates the liquid region from the solid deposit. *r*_r_(*t*) and *r*_or_(*t*) are the radii of the radial and orthoradial crack propagation fronts, respectively. Because water cannot flow past an orthoradial crack, the velocity *u*(*r*, *t*) at *r*_or_ is zero. (**B**) Spatiotemporal evolution of the pore pressure in the deposit scaled by the pressure scale $P^{*}=\left(\mu jr_{0}^{2}\right)/\left(k\bar{h}\right)$ , calculated using Eq. 1 with the measured deposit thickness and orthoradial crack front. The pressure decreases spatially away from the liquid region and decreases with time. The pressure at the boundary *r*_l_ with the liquid region, indicated by circles, is small compared to P*. The pressure at the radial crack front is indicated by diamonds; the pressure at the orthoradial crack front is indicated by squares. (**C**) Pore pressure in the deposit at the crack propagation fronts scaled by P*, multiplied by the square root of the deposit thickness scaled by the average deposit thickness $\bar{h}$ . The value of $P/P^{*}\sqrt{h/\bar{h}}$ at the radial crack front (diamonds) remains constant for *r*/*r*_0_ < 0.6, while the value at the orthoradial crack front (squares) decreases as the front propagates to smaller radii.

The radial flow of water from the liquid region through the deposit is sketched in Fig. 4A. Because the deposit is thin (*h* ≪ *r*_0_), the radial flow velocity is much greater than the vertical flow velocity (*34*). The height-averaged radial water velocity *u*(*r*, *t*) satisfies ∂(*uhr*)/∂*r* + *jr* = 0, where *j* is the evaporative flux that we assume to be constant (*34*, *35*). The viscous dissipation associated with water flow in the pores of the deposit leads to a pressure gradient obtained from Darcy’s law, ∂*P*/∂*r* = − μ*u*/*k*, where *P*(*r*, *t*) is the water pore pressure, μ is the dynamic viscosity of water, and *k* is the permeability of the deposit, which is proportional to the particle size squared (*6*, *11*, *33*, *36*–*57*). The flow velocity inside the deposit is maximum at the edge of the liquid region and reaches zero at the orthoradial crack front, *u*(*r*_or_) = 0, because water cannot flow across an orthoradial crack. Accordingly, the pressure decreases with *r* and is maximally negative at the orthoradial crack front. Combining Darcy’s law with mass conservation and the measured deposit thickness *h*(*r*) yields

$$P\left(r,t\right)=P\left(r_{l}\right)-\frac{\mu jr_{0}^{2}}{k\bar{h}}\int_{r_{l}\left(t\right)}^{r}‍\frac{\bar{h}}{2r_{0}^{2}}\frac{r_{\text{or}}^{2}\left(t\right)-r^{\prime 2}}{h\left(r\prime \right)r\prime }\mathrm{dr}\prime$$ (1)

This expression features the porous pressure scale, $P^{*}=\left(\mu jr_{0}^{2}\right)/\left(k\bar{h}\right)$ with $\bar{h}$ an average deposit thickness, multiplied by a term that captures the decrease in pressure with *r*. A smaller radius *r*_l_ of the solidification front or a larger radius *r*_or_ of the orthoradial crack front decrease the pressure. Because of the low permeability *k* ≪ *h*^2^ of the deposit, P* is of the order of 1 to 10 MPa, while the pressure at the solid-liquid boundary *P*(*r*_l_) ≪ P*; we thus neglect *P*(*r*_l_) in the pressure calculations (*36*). The pressure profiles obtained using the measured values of *h* and *r*_or_ for an experiment at ϕ_0_ = 0.05 are shown in Fig. 4B.

We can now understand the rapid decrease in the size of the liquid region at the end of drying observed in the last 5 s of Fig. 3C as a consequence of the water transport from the liquid region to the solid deposit. For water from the liquid region to replace all the water that evaporates from the top of the deposit between *r*_l_ and *r*_or_, the water flux out of the liquid region 2π*r_l_u*(*r*_l_)*h*(*r*_l_) must be equal to the water flux out of the deposit $\pi j\left(r_{\text{or}}^{2}-r_{l}^{2}\right)$ . As the liquid region shrinks, the water velocity out of the liquid region at *r*_l_ increases and eventually diverges as $u\left(r_{l}\right)∼jr_{\text{or}}^{2}/\left(2hr_{l}\right)$ , accelerating the retraction of the liquid region (*36*). These faster flows cause the pressure to decrease faster with *r*, causing the radial cracks to propagate closer to the liquid region as observed for *t* > 140 s in Fig. 3C.

How does the pore pressure in the deposit govern the location of the radial crack front? Because of the negative pressure in the deposit, the deposit wants to shrink. However, it is attached to the substrate and cannot deform in the in-plane directions. As a result, an in-plane misfit tensile stress σ_0_ = − *P*(1 − 2ν)/(1 − ν) develops in the deposit, where ν is the Poisson ratio of the deposit (*6*). The misfit stress is the stress in the film in the absence of cracks; once cracks form, the stress decreases compared to the misfit stress. This misfit stress increases as the pressure decreases and eventually causes cracks to form and propagate. The propagation of the radial cracks is set by a balance between the elastic energy released and the energy cost for forming a fracture, which includes the surface energy cost of creating new surfaces as well as inelastic deformation costs (*37*, *58*). An isolated crack in a thin film releases stresses in the direction perpendicular to the crack over a distance proportional to the deposit thickness *h* (*51*, *59*, *60*). The elastic energy released by the crack propagating over a length *dl* scales as the elastic energy density times the volume of deposit where stresses are released: $\sigma_{0}^{2}/E\cdot h^{2}\mathrm{dl}$ , where *E* is the Young’s modulus of the deposit (*60*, *61*). The fracture energy cost is equal to *G*_c_*hdl*, where *G*_c_ is the critical energy release rate (*62*). Balancing the two terms yields a dimensionless load parameter

$$\Sigma =\sigma_{0}\sqrt{\frac{h}{EG_{c}}}=-P\frac{1-2\nu }{1-\nu }\sqrt{\frac{h}{EG_{c}}}$$ (2)

that quantifies the balance between elastic energy and fracture energy (*54*). Σ quantifies the propensity of the deposit to fracture and increases with the misfit stress set by the pore pressure σ_0_ ∼ − *P*, the deposit thickness *h*, and the brittleness of the deposit (*EG*_c_)^−1^. An isolated crack propagates when Σ = Σ_c_, where Σ_c_ is a constant of order unity (*54*, *60*). Preexisting cracks lower the stress σ in the deposit compared to the misfit stress σ_0_, decreasing the elastic energy released by a potential new crack and requiring values of Σ larger than the isolated single crack value of Σ_c_ to form additional cracks.

As shown in Fig. 4C, the radial cracks propagate at a constant value of $P/P^{*}\sqrt{h/\bar{h}}∼-\Sigma$ for *r*/*r*_0_ < 0.6. Radial cracks propagate in a region of the deposit that has no preexisting cracks, and with a relatively large spacing between the cracks compared to the deposit thickness, thus we assume that the energy release rate for radial crack propagation is close to the energy release rate for an isolated crack and that Σ ≈ Σ_c_. By contrast, orthoradial cracks form further away from the liquid region, requiring more negative pressures and a larger load parameter Σ compared to radial cracks. This indicates that the formation of an orthoradial crack between two radial cracks releases less elastic energy than the propagation of a radial crack in a region with no preexisting cracks. An orthoradial crack requires a larger load parameter not only because of the orientation of the crack but also because it forms after radial cracks have already partially released the stresses in the deposit. In addition, the load parameter Σ required for forming new orthoradial crack at the orthoradial crack front continuously increases, as shown in Fig. 4C, indicating that orthoradial cracks forming later at smaller radii release less elastic energy than orthoradial cracks forming at early times closer to the deposit edge. This might be due to the deposit shape that sets the final distribution of orthoradial cracks, as shown in Fig. 3D.

The formation of orthoradial cracks disrupts the radial flow of water in the deposit. Water that evaporates from the deposit at *r* > *r*_or_ can no longer be replenished by water from the liquid region, and air invades the deposit. When the deposit is still saturated with water, the pore pressure inside the deposit is balanced with atmospheric pressure at the microscopic water-air menisci via the capillary pressure *P*_atm_ − *P*(*r*, *t*) ∼ γκ(*r*, *t*), where γ is the water-air surface tension and κ is the curvature of the menisci (*35*, *36*, *56*). The menisci can sustain a pressure difference between air and water in the pores of up to *P*_atm_ − (−*P*_cap_) ≈ 5γ/*a* = 7.4 MPa. Air starts to invade the deposit when the pressure reaches *P* = −*P*_cap_. Air invasion is visible in the microscope images because the deposit is transparent when saturated with water or air but appears optically opaque when air partially invades (*15*, *36*). In the seconds following the formation of an orthoradial cracks, the fragment of deposit created behind the orthoradial crack becomes opaque and later transparent again, as shown in Fig. 3 (A and B) and in movie S2.

### Densification of radial cracks

**Fig. 5.**
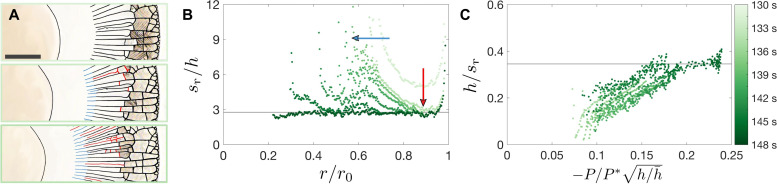
Temporal evolution of radial cracks. (**A**) Section of a 0.3-μl, ϕ_0_ = 0.05 suspension drop at *t* = 137 s (top), *t* = 139 s (middle), and *t* = 141 s (bottom) after deposition. The propagation of radial cracks is highlighted in blue, and the formation of new radial cracks between preexisting cracks is highlighted in red. The scale bar represents 250 μm. (**B**) Ratio of the radial crack spacing and deposit thickness *s*_r_/*h* versus *r*/*r*_0_ at different times since drop deposition. Over time, the *s*_r_/*h* curves extend to smaller values of *r*/*r*_0_ because of radial crack propagation (blue arrow) and shift downward because of additional radial crack formation (red arrow). (**C**) Normalized crack density *h*/*s*_r_ versus $-P/P^{*}\sqrt{h/\bar{h}}∼\Sigma$ ; the data at different times collapse onto a master curve. The gray horizontal lines in (B) and (C) correspond to the constant values of *s*_r_/*h* and *h*/*s*_r_ of the final crack pattern for this experiment.

As the deposit grows, the radial cracks propagate at a distance *r*_r_ from the center of the drop, as highlighted in blue in Fig. 5A. This crack propagation front, however, does not capture the entire dynamics of the radial crack formation: Additional radial cracks form in between existing cracks, as highlighted in red in Fig. 5A and in movie S3. As a consequence, the radial crack spacing *s*_r_ measured at the radial crack propagation front is larger than the final radial crack spacing. To quantify this dynamic evolution of the radial crack spacing over time and space, we measure *s*_r_(*r*, *t*) with a time step of 1 s, using an algorithm described in fig. S3. The radial crack spacing divided by the local deposit thickness indeed decreases with time as radial cracks propagate and additional radial cracks form, as shown in Fig. 5B. The propagation of existing radial cracks extends the curves of *s*_r_/*h* to the left toward smaller values of *r*, while the formation of additional radial cracks shifts the curves of *s*_r_/*h* downward toward the constant value *s*_r_/*h* = 3.1 ± 0.7 measured for the final crack pattern.

We rationalize this dynamics of the radial crack spacing considering that the formation of the first generation of radial cracks at Σ = Σ_c_ partially releases the stresses in the deposit. Consequently, a new radial crack forming at *r* > *r*_r_ (shown in red in Fig. 5A) will release less elastic energy than a propagating radial crack at *r*_r_ (shown in blue), while the fracture energy cost remains the same; a new radial crack thus forms for a higher dimensionless load Σ. As the deposit thickness sets the distance over which stresses are released by a crack, the fraction of the elastic energy available for additional crack formation depends on the crack spacing scaled by the local deposit thickness, *s*_r_/*h* (*50*, *54*). Confirming this rationale, the normalized crack density *h*/*s*_r_ collapses onto a master curve when plotted against $-P/P^{*}\sqrt{h/\bar{h}}∼\Sigma$ , as shown in Fig. 5C. The entire dynamics of radial crack propagation and densification can thus be captured by a balance between elastic energy due to a decrease in pore pressure, fracture energy, and energy released by previous cracks. For Σ < Σ_c_, no crack can form. The propagation of the first generation of radial cracks, which are spaced far apart and do not interact with each other, occurs at Σ = Σ_c_, which corresponds to $-P/P^{*}\sqrt{h/\bar{h}}\approx 0.1$ in Fig. 5C. For Σ > Σ_c_, the crack density *h*/*s*_r_ increases until it reaches the constant *h*/*s*_r_ that is also observed when measuring the final crack density. A similar increase has previously been reported for the case of a film of constant thickness under an increasing misfit stress (*27*). Here, we address the much more complex case of a deposit of nonuniform thickness with a misfit stress that varies in space and time as a solidification front propagates and orthoradial cracks stop the flow.

### A master curve for transient and final crack density

We have seen that air invasion occurs when the pore pressure in the deposit reaches *P* = −*P*_cap_. This pressure is reached either after the deposit is isolated from the liquid region by an orthoradial crack or when the liquid region shrinks to a small size, decreasing the pressure. The onset of air invasion corresponds to the maximum value of the dimensionless load parameter reached during the drying process, $\Sigma_{f}=\left(1-2\nu \right)/\left(1-\nu \right)\;P_{\text{cap}}\sqrt{h/\left(EG_{c}\right)}$ . The constraint that Σ_f_ has to be greater than Σ_c_ for a crack to form provides an expression for the critical deposit thickness below which cracks do not form $h_{c}={\left(\frac{1-\nu }{1-2\nu }\right)}^{2}\frac{\Sigma_{c}^{2}EG_{c}}{P_{\text{cap}}^{2}}$ : Cracks do not release enough elastic energy to propagate through thin regions of the deposit where *h* < *h*_c_ (*59*, *60*). We measure *h*_c_ = 0.8 μm, which assuming ν = 0.3 and Σ_c_ = 1 provides an estimate for the fracture toughness $\sqrt{EG_{c}}=3.8\times 10^{3}\;\mathrm{Pa}\;m^{1/2}$ , a value that is in good agreement with that obtained by measuring the shape of the crack tip (*58*).

**Fig. 6.**
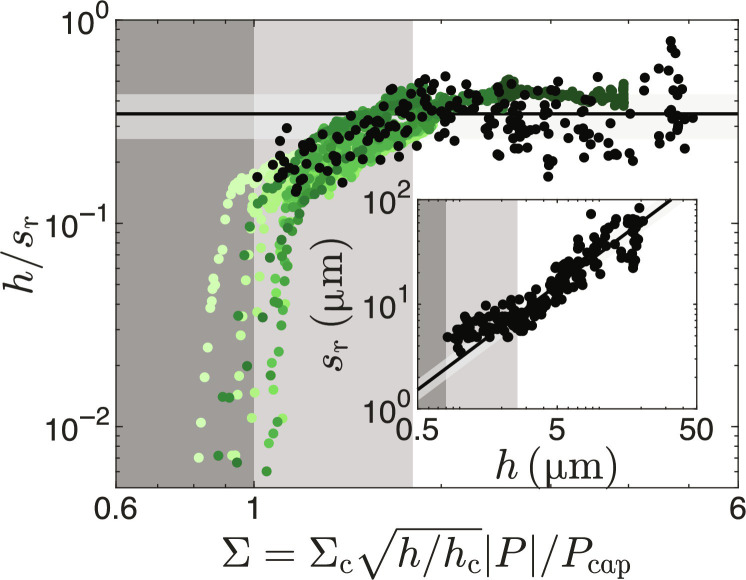
Increase and saturation of crack density. The normalized radial crack density *h*/*s*_r_ increases as a function of the dimensionless load parameter Σ and saturates at *h*/*s*_r_ = 0.35 ± 0.09 for Σ > 1.8. For the dynamic crack spacing data, shown in shades of green, Σ varies with both *h* and ∣*P*∣. For the final crack spacing data, shown in black, ∣*P* ∣ /*P*_cap_ = 1 and Σ depends only on *h*. The dark shaded area indicates Σ < Σ_c_, and the lighter shaded area indicates Σ_c_ < Σ < Σ_sat_; the horizontal line and shaded area indicate *h*/*s*_r_ = 0.35 ± 0.09. Inset: The local deposit thickness and the final radial crack spacing are directly measured for individual crack fragments. No cracks form for *h* < 0.8 μm, as indicated by the dark shaded area. The crack spacing deviates from a linear scaling with deposit thickness for *h* < 2.6 μm, as indicated by the lighter shaded area. The diagonal line and shaded area correspond to *s*_r_ = (3.1 ± 0.7)*h*.

Additional cracks form and decrease the crack spacing as Σ increases from Σ_c_ up to its maximum value $\Sigma_{f}=\Sigma_{c}\sqrt{h/h_{c}}$ that depends on the local deposit thickness *h*. Simulations have shown that for a sufficiently small ratio of the crack spacing over deposit thickness, additional cracks release no elastic energy and crack formation stops (*25*, *52*). The crack spacing is then proportional to the deposit thickness. This regime of saturated crack density is reached for a dimensionless load Σ_sat_ and corresponds to the ratio *s*_r_/*h* = 3.1 ± 0.7 measured for the final crack patterns. However, in the range Σ_c_ < Σ_f_ < Σ_sat_, which corresponds to *h*_c_ < *h* < *h*_sat_ = *h*_c_(Σ_sat_/Σ_c_)^2^, we expect the final crack spacing to be larger than (3.1 ± 0.7)*h* and to decrease with $\Sigma_{f}\propto \sqrt{h}$ . Indeed, by directly measuring the local crack spacing and the deposit thickness for individual dried deposit fragments (inset of Fig. 6), we observe that *s*_r_ is larger than (3.1 ± 0.7)*h* for *h* < *h*_sat_ = 2.6 μm corresponding to Σ_f_ < Σ_sat_ = 1.8, as shown in Fig. 6. Thin regions of the deposits where *h* < *h*_sat_ are less prone to cracking such that air invades the deposit before the crack density saturates. These regions occur toward the center of the deposit, and the cracks there are smaller than the image resolution and are not captured in our crack analysis algorithm, which explains why the radial crack spacing is proportional to the thickness for the entire range shown in Fig. 2C.

To compare this dependence of *h*/*s*_r_ on Σ observed in the thin final deposit fragments with that obtained from the dynamics, we calculate Σ in the dynamics. To do so, we assume that Σ(*r*_r_) = Σ_c_ at the radial crack propagation front, which implies that $-P\left(r_{r}\right)/P^{*}\sqrt{h\left(r_{r}\right)/h_{c}}=P_{\text{cap}}/P^{*}\approx 0.3$, where the value is calculated by normalizing the data shown in Fig. 4C by $\sqrt{\bar{h}/h_{c}}=3$. This yields the dimensionless load parameter

$$\Sigma =\Sigma_{c}\frac{-P}{P_{\text{cap}}}\sqrt{\frac{h}{h_{c}}}$$ (3)

where *P*/*P*_cap_ is calculated as (*P*/P*)(P*/*P*_cap_). Σ is thus obtained from the measured data and Σ_c_ ≈ 1, without needing to estimate the elastic properties of the deposit, *P*_cap_, or P*. The data of *h*/*s*_r_ = *f*(Σ) obtained from the dynamics measurement are in excellent agreement with those obtained by measuring the final crack spacing in thin fragments for experiments at all volume fractions, indicating that the balance between elastic energy, fracture energy, and energy released by previous cracks governs both the dynamic densification of radial cracks where Σ depends on both *P* and *h* and the final crack spacing where Σ = Σ_f_ depends only on *h*.

## DISCUSSION

By drying drops of a colloidal suspension and measuring the resulting deposit thickness and crack spacing, we show that the spacing between cracks oriented in the radial direction is set by the local deposit thickness, while the spacing between cracks oriented in the orthoradial direction depends on both the local deposit thickness and the deposit shape. To capture the complex dynamics of crack formation, we couple poroelasticity with fracture mechanics. We establish a model for the pore pressure in the deposit that takes into account the local deposit thickness and the propagating solidification and orthoradial crack fronts. The negative pore pressure in the deposit coupled with adhesion of the deposit to the substrate causes a tensile misfit stress in the deposit, which leads to crack formation. A crack forms when the elastic energy released by a crack is larger than the energetical cost of fracture, a balance quantified by the dimensionless load parameter Σ that depends on the misfit stress, on the deposit thickness and on the brittleness of the deposit. As a first generation of radial cracks releases a portion of the stress, a larger misfit stress is required to create additional cracks in the same deposit segment. Despite the spatially varying deposit thickness and pore pressure, the densification of radial cracks only depends on the local deposit thickness and the load, resulting in a master curve relating the dimensionless radial crack density *h*/*s*_r_ to the dimensionless load parameter Σ. Both the propagation of radial cracks and the formation of additional radial cracks behind the propagation front are captured by this master curve. In addition, because air eventually invades the deposit and air invasion occurs at a fixed pressure −*P*_cap_, the final radial crack spacing can be cast onto the same master curve where the dimensionless load parameter Σ depends only on the deposit thickness *h*. This master curve presents two regimes. At small loads, the radial crack density increases with the load, and consequently, the final radial crack density increases with *h* for small *h*. At larger loads Σ > 1.8, the radial crack density saturates at *h*/*s*_r_ = 0.35 ± 0.09, yielding the constant ratio of local crack spacing over local deposit thickness measured for most of the dried deposit.

One-dimensional models of crack formation in elastic films predict that the ratio between the film thickness and the crack spacing increases without bounds as the stress increases (*25*), in disagreement with experiments in films of uniform thickness that reveal a saturation of the ratio (*27*, *63*, *64*). Two different mechanisms have been proposed to account for this saturation. The first mechanism rationalizes that as the aspect ratio of a crack segment *s*_r_/*h* becomes smaller, the elastic problem transitions from one-dimensional to two-dimensional. Because of the deformation of the deposit around a crack, stresses become compressive in the center of the film (*25*, *50*). Additional crack formation is prevented because the energy release rate of a new crack becomes negative, leading to a constant ratio of film thickness over crack spacing that depends on the elastic properties of the film and of the substrate (*52*). The second mechanism considers that the film can debond from the substrate at the cost of an adhesion energy per unit area $G_{c}^{\text{debond}}>G_{c}$ (*53*, *54*). For large enough dimensionless loads Σ, debonding becomes more favorable than forming additional cracks. A further increase in the load leads to more debonding, and the ratio of crack spacing over deposit thickness saturates at a value that only depends on the ratio $G_{c}/G_{c}^{\text{debond}}$ (*54*). Such debonding, or delamination, occurs in many drying deposits (*20*, *42*, *47*, *65*). We use interferometric imaging to probe whether debonding around cracks occurs in our experiments. As shown in fig. S4, debonding is only visible for a limited number of crack fragments, suggesting that debonding is not the main mechanism for crack saturation.

We observe that orthoradial cracks form with a smaller spacing in the bump region of the deposit compared to the center of the deposit, as shown in Fig. 2D. The critical energy release rate *G*_c_ is the same for all cracks, but the energy release rate can be different for each crack. The variation in the orthoradial crack spacing thus suggests that the shape of the deposit affects the energy release rate of orthoradial cracks but not of radial cracks. The dimensionless load at the orthoradial crack front continuously increases during drying, as shown in Fig. 4C, indicating that forming orthoradial cracks at smaller radii (and outside the bump region of the deposit) releases progressively less elastic energy. The shape of the deposit has been related to the direction of the stress in the absence of cracks in recent work (*49*), but an energetic approach is needed to capture the cumulative effect of the deposit shape and of the preexisting radial cracks on orthoradial crack formation (*16*).

Orthoradial cracks provide an interesting example of a two-way coupling between the pore pressure, which sets the misfit stress, and crack formation. The misfit stress is the stress that the material would experience in the absence of cracks and is thus by definition typically not affected by cracks. However, because orthoradial cracks prevent the radial flow of water inside the deposit, an orthoradial crack changes the pore pressure and, in turn, the misfit stress in the deposit.

Our model for the local stress in the deposit captures the propagation and densification of the radial cracks and extends previous pore pressure models by accounting for the varying deposit thickness and for the location of the solidification and orthoradial crack fronts (*35*, *36*). To obtain the pore pressure and subsequently the tensile stress in the deposit, we use key simplifying assumptions that we would like to briefly address. Relaxing these assumptions could provide avenues for future work. (i) To describe the flow in the deposit, we assume that the evaporative flux *j* is uniform over the water-saturated deposit for *r* < *r*_or_. The evaporative flux is set by the diffusion of water vapor away from the surface of the deposit and is expected to be larger closer to the edge of the deposit where water vapor can diffuse away from the deposit in more directions (*66*). The propagation of a dry deposit region for *r* > *r*_or_, however, adds intricacies to estimating the evaporative flux. (ii) To estimate the pressure *P* in Eq. 1, we assume that the permeability *k* of the deposit is uniform and constant. In reality, *k* depends on the volume fraction of the deposit, which increases away from the solidification front and might also increase with time as the pore pressure decreases (*6*, *35*, *49*, *55*). (iii) We estimate the misfit stress σ_0_ = − *P*(1 − 2ν)/(1 − ν) by assuming a linear stress-strain relation with uniform Young’s modulus and Poisson ratio. Like the permeability, the elastic properties of the deposit depend on the volume fraction of the deposit (*58*). Furthermore, the linear stress-strain relationship does not fully capture the mechanics of particle deposits, and a nonlinear stress-strain relation has been proposed on the basis of spherical particles in Hertzian contact (*67*). This model provides an expression for the elastic properties of the deposit that depends on the elastic properties of the particle and on the volume fraction of the deposit, and captures the effect of a crack opening on the local pressure (*68*). (iv) The deposit shrinks slightly upon the formation and further opening of a crack. If both water and particles are incompressible, this shrinkage corresponds to a local increase in deposit volume fraction accomplished by the expulsion of water, creating a temporary flow away from the crack inside the deposit and a local increase in pore pressure (*69*). This flow is small compared to the radial flow due to evaporation but could induce additional interactions between adjacent cracks.

Complementary to refining the modeling, experiments that directly measure the local stress in the drying deposit could provide additional insight into the relation between drying stress and crack propagation. The stress in the deposit can be estimated by depositing the suspension on a flexible membrane or on an elastomer of known elastic properties (*70*–*73*). Given the sensitivity of the crack pattern on the surface and elastic properties of the substrate, however, these experiments are challenging and would require great care in establishing a correspondence between different experimental conditions (*19*). Our demonstration that the crack density is set dynamically and locally by the pore pressure opens pathways to using measurements of the dynamic crack spacing to infer stresses in drying thin films, while the final crack spacing could be used to estimate the film thickness.

## MATERIALS AND METHODS

Experiments are performed using charge-stabilized suspensions of carboxylated polystyrene particles (CA100NM, MagSphere) with particle diameter 2*a* = 97 ± 15 nm. The suspensions are diluted with deionized water to achieve particle volume fractions in the range ϕ_0_ = 0.03 to 0.10. The suspensions remain stable after dilution. Drops of volume 0.3 μl are deposited on clean microscope glass slides using a micropipette (Gilson 0.2-2 μl). The drop contact angle right after deposition is (18 ± 2)°. Before use, the glass slides are cleaned by sonication in acetone for 5 min, rinsed with isopropanol, and dried with pressurized air. The colloidal suspensions undergo 20 min of sonication to ensure complete dispersion. The drops are placed in a transparent box to minimize air flow and dry at an ambient temperature of *T* = (22 ± 2)°C and a constant relative humidity ranging between 25 and 52%. We image the drops from below using an inverted optical microscope (Eclipse TE2000-U, Nikon, equipped with a Lumix GH5 camera) at ×4 magnification, filming at 24 fps. After drying is completed, we measure the deposit thickness using a laser scanning confocal microscope (VK-X 3D, Keyence) with a ×20 magnification objective. Cracks are identified in the microscope image of the final dried deposit using a local thresholding algorithm (*74*) followed by skeletonization and use of the ImageJ Analyze Skeleton plugin (*75*). Dynamic crack identification is performed by following the same steps on a series of difference images, taking care to only count each crack once, as shown in fig. S3.
