# Supplementary material for: Crack densification in drying colloidal suspensions
Source: Sci Adv. 2024 Sep 11;10(37):eadp3746. doi: 10.1126/sciadv.adp3746 (PMC11389785; doi:10.1126/sciadv.adp3746)
Supplement: Supplementary file 1 — Supplementary Text Figs. S1 to S4 Legends for movies S1 to S3 [file sciadv.adp3746_sm.pdf]

Science Advances
AAAS

Supplementary Materials for

Crack densification in drying colloidal suspensions

Paul Lilin *et al.*

Corresponding author: Irmgard Bischofberger, irmgard@mit.edu

Sci. Adv. **10**, eadp3746 (2024)
DOI: 10.1126/sciadv.adp3746

The PDF file includes:

Supplementary Text
Figs. S1 to S4
Legends for movies S1 to S3

Other Supplementary Material for this manuscript includes the following:

Movies S1 to S3

S1. Classification of radial and orthoradial cracks

The orientation of the cracks is set by the circular drop geometry: Cracks orient preferentially along either the radial or the orthoradial direction with regard to a point in the center of the drop that we refer to as the center of drying. Because radial and orthoradial cracks display distinct dynamics and crack spacings, we analyze them separately. To classify a crack, we locate its two endpoints (r_1, θ_1) and (r_2, θ_2) in a polar coordinate system centered at the center of drying. We calculate the coordinates of the center of the crack $r = (r_1 + r_2)/2$ and $\theta = (\theta_1 + \theta_2)/2$ and the extent of the crack $\Delta r = r_2 - r_1$ and $\Delta\theta = \theta_2 - \theta_1$, as shown in Fig. S1a. The extent of the crack in pixels in the orthoradial direction is $r\Delta\theta$. The result of the crack analysis and classification is shown in Fig. S1b, where cracks with $\Delta r > 1.1 r\Delta\theta$ are classified as radial and cracks with $r\Delta\theta > 1.1\Delta r$ are classified as orthoradial. This classification criterion is shown in Fig. S1c.

To obtain the analyzed cracks from the original image, we increase the contrast of the original image and use a local thresholding algorithm *(65)*. We clean up the thresholded image by removing thresholded regions with an area lower than a set value and by performing a binary closure operation. We then skeletonize the image and use the ImageJ Analyze Skeleton plugin to obtain a list of crack endpoints *(66)*.

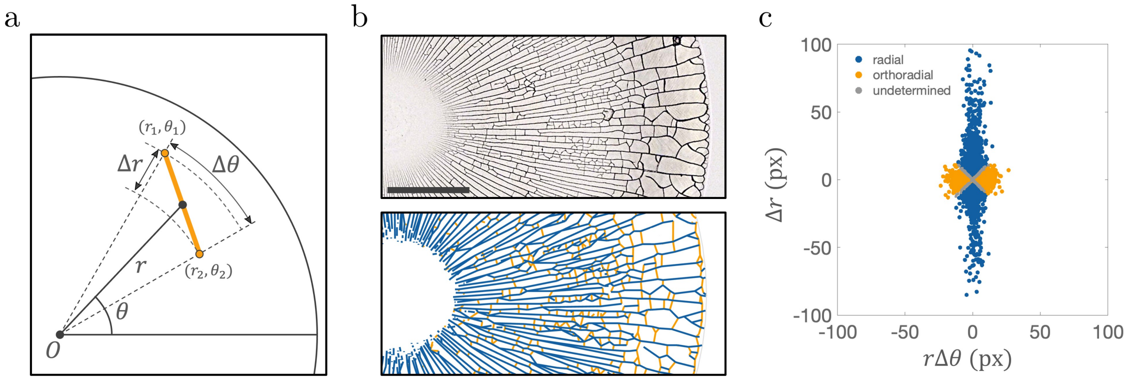

Fig. S1. Classification of radial and orthoradial cracks. (a) Definition of the coordinate system for a single crack, shown in orange. O marks the center of drying. The polar coordinates (r, θ) quantify the location of the center of the crack, and $(\Delta r, \Delta\theta)$ quantifies the extent of the crack. The extent of the crack in the orthoradial direction in pixels is $r\Delta\theta$. (b) Final crack pattern for a drop with initial particle volume fraction $\phi_0 = 0.05$. Top: Microscope image of the final crack pattern. The scale bar represents $250\,\mu\mathrm{m}$. Bottom: Analyzed crack pattern with cracks classified as radial shown in blue and cracks classified as orthoradial shown in orange. (c) Crack extent in the radial direction Δr *versus* crack extent in the orthoradial direction $r\Delta\theta$ for each crack in (b). Cracks with $\Delta r > 1.1 r\Delta\theta$ are classified as radial, cracks with $r\Delta\theta > 1.1\Delta r$ are classified as orthoradial. The remaining cracks (less than 4% of the total number of cracks) are undetermined and not used in the subsequent analysis.

S2. Measurement of radial and orthoradial crack spacings

The spacing between radial cracks and between orthoradial cracks varies across the deposit. For both radial and orthoradial cracks, the crack spacing is defined in the direction perpendicular to the crack orientation, as shown in Fig. S2. For a pair i, j of radial cracks with centers located at (r_i, θ_i) and (r_j, θ_j) as defined in section S1, we define the local radial crack spacing at radius r as $s_r^\theta = r|\theta_i - \theta_j|$. Conversely, we define the local orthoradial crack spacing between a pair of orthoradial cracks as $s_{or}^\theta = |r_i - r_j|$. We scale the radial coordinates r of each crack by the corresponding radius $r_0(\theta)$ from the center of drying to the edge of the deposit passing through the crack, thus scaling the crack pattern to fit into a circle of radius unity centered on the center of drying. Because of the polar symmetry of the drops, we average the measured values of s_r^θ and s_{or}^θ at a given scaled radius r/r_0.

To measure the radial crack spacing at radius r/r_0, we draw a circle of radius r/r_0 and identify the radial cracks that cross this circle, as shown in Fig. S2a. By calculating the local spacing s_r^θ between each pair of adjacent cracks, we obtain the polar-angle averaged radial crack spacing s_r and the standard deviation of the s_r^θ distribution, as shown in Fig. S2b. We repeat this process at each r to obtain the data shown in Fig. S2c.

To measure the orthoradial crack spacing at radius r, we draw a circle of radius r centered at the center of drying and identify the orthoradial cracks closest to this circle, as shown in Fig. S2d. We then measure the orthoradial crack spacing s_{or}^θ for each angle θ by drawing a line at angle θ and identifying the pair of orthoradial cracks that cross the line. By repeating this measurement with an increment of 1°, we obtain the polar-angle averaged orthoradial crack spacing s_{or} and the standard deviation of the s_{or}^θ distribution, as shown in Fig. S2e. We repeat this process at each r to obtain the data shown in Fig. S2f.

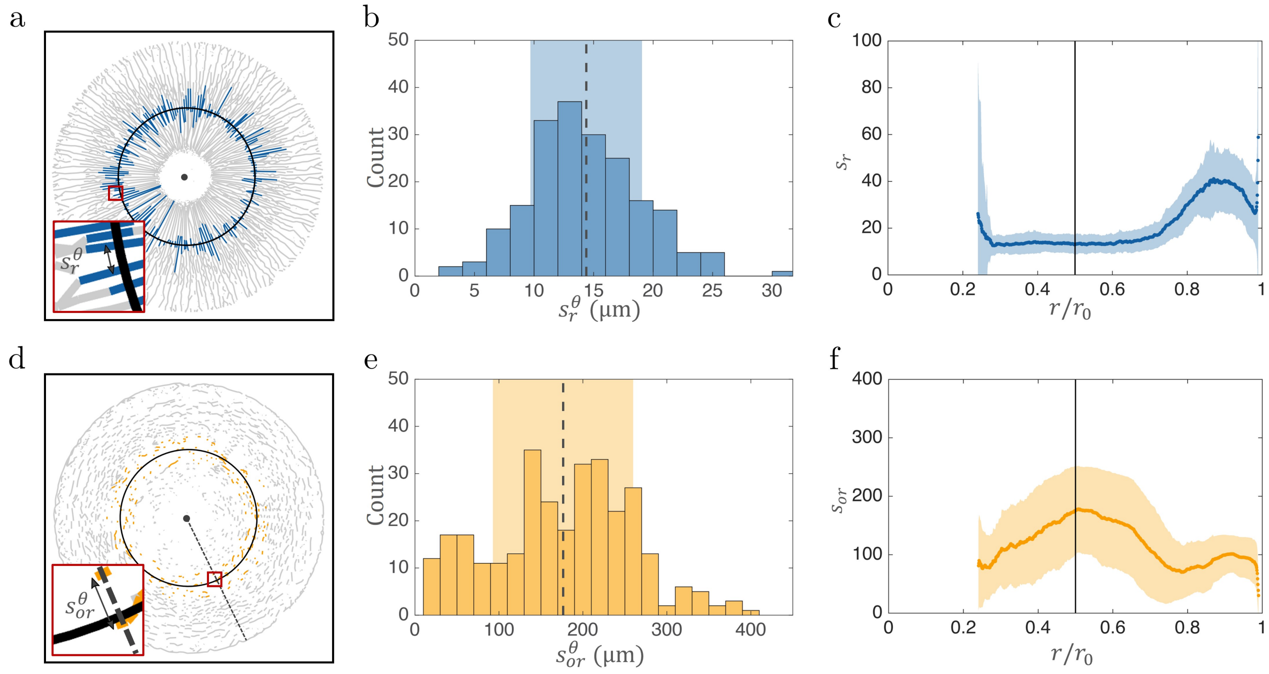

Fig. S2. Radial and orthoradial crack spacing. (a) Final radial crack pattern. Radial cracks that cross the circle of radius $r/r_0 = 0.5$ are highlighted in blue. We measure the local spacing s_r^θ between adjacent radial cracks. (b) Histogram of the local radial crack spacing s_r^θ at $r/r_0 = 0.5$. The dashed line indicates the $\theta-$averaged radial crack spacing $s_r = \left\langle s_r^\theta \right\rangle$ and the shaded area indicates the standard deviation $\sigma_{s_r^\theta}$. (c) Radial crack spacing s_r and standard deviation $\sigma_{s_r^\theta}$ (shaded region) measured at each radius r/r_0. (d) Final orthoradial crack pattern. Orthoradial cracks that are closest to the circle of radius $r/r_0 = 0.5$ are highlighted in orange. The local orthoradial crack spacing s_{or}^θ is defined at each polar angle θ by drawing a line at θ and identifying the closest orthoradial cracks on both sides of the circle that cross the θ line, as shown in the inset. (e) Histogram of the local orthoradial crack spacing s_{or}^θ at $r/r_0 = 0.5$. We perform 360 measurements at increasing θ values. The dashed line indicates the $\theta-$averaged orthoradial crack spacing $s_r = \left\langle s_r^\theta \right\rangle$ and the shaded area indicates the standard deviation $\sigma_{s_{or}^\theta}$. (f) Orthoradial crack spacing s_{or} and standard deviation $\sigma_{s_{or}^\theta}$ (shaded region) measured at each radius r/r_0.

S3. Time-resolved crack identification

Dynamic crack identification is performed by analyzing difference images between the images $(f_i)_{1,\ldots,n}$ saved at a one second interval. We calculate the 8-bit absolute difference images d_i between images f_{i+1} and f_i, as shown in Fig. S3a. A pixel can change color between f_i and f_{i+1} for three reasons: a crack can form which is optically opaque and darkens the pixel; the solidification front that separates the liquid region and solid deposit can pass through the pixel and change its color; and the pixel can become darker due to air invasion in the solid deposit. Our aim is to retain the changes induced by crack formation and remove the other two sources of change. To do so, we first manually remove the solidification front from the difference i mages. Removing air invasion is more difficult and requires two steps. First, we apply a spatial filtering s tep s hown i n Fig. S 3b. We t hreshold t he difference images and conduct a pixel-wise AND operation between each thresholded difference image $\hat{d}_i$ and the thresholded final c rack p attern $\hat{f}_n$ to o btain ($m_i)_{1,\ldots,n}$. T his e nsures t hat o nly p ixels pertaining to cracks are present. However, a given location might change intensity first due to crack formation and later due to air invasion, thus appearing twice in $(m_i)_{1,\ldots,n}$ and leading to the double-counting of cracks. We solve this problem by only keeping the first instance that a pixel appears in $(m_i)_{1,\ldots,n}$, performing a temporal filtering step shown in Fig. S 3c. We reverse the stack of images to $(m_i)_{n\ldots1}$, then flatten it into a single 2-dimensional image where the intensity value of a pixel encodes the last index i at which the pixel appears in $(m_i)_{n\ldots1}$. Because the stack is reversed, this index corresponds to the first time the pixel appears, which corresponds to crack formation. The single image is converted back to a stack of binary images $(m^*_i)_{n\ldots1}$. The binary images are skeletonized, reducing the width of each crack to a single pixel. We then use the ImageJ Analyze Skeleton plugin *(66)* on each skeletonized image to obtain the endpoints of each crack, and classify each crack according to the criteria discussed in section S1. In Fig. S3d, we sum the analyzed images $(a_i)_{1,\ldots,n}$ to recover all the cracks that have formed up to time i.

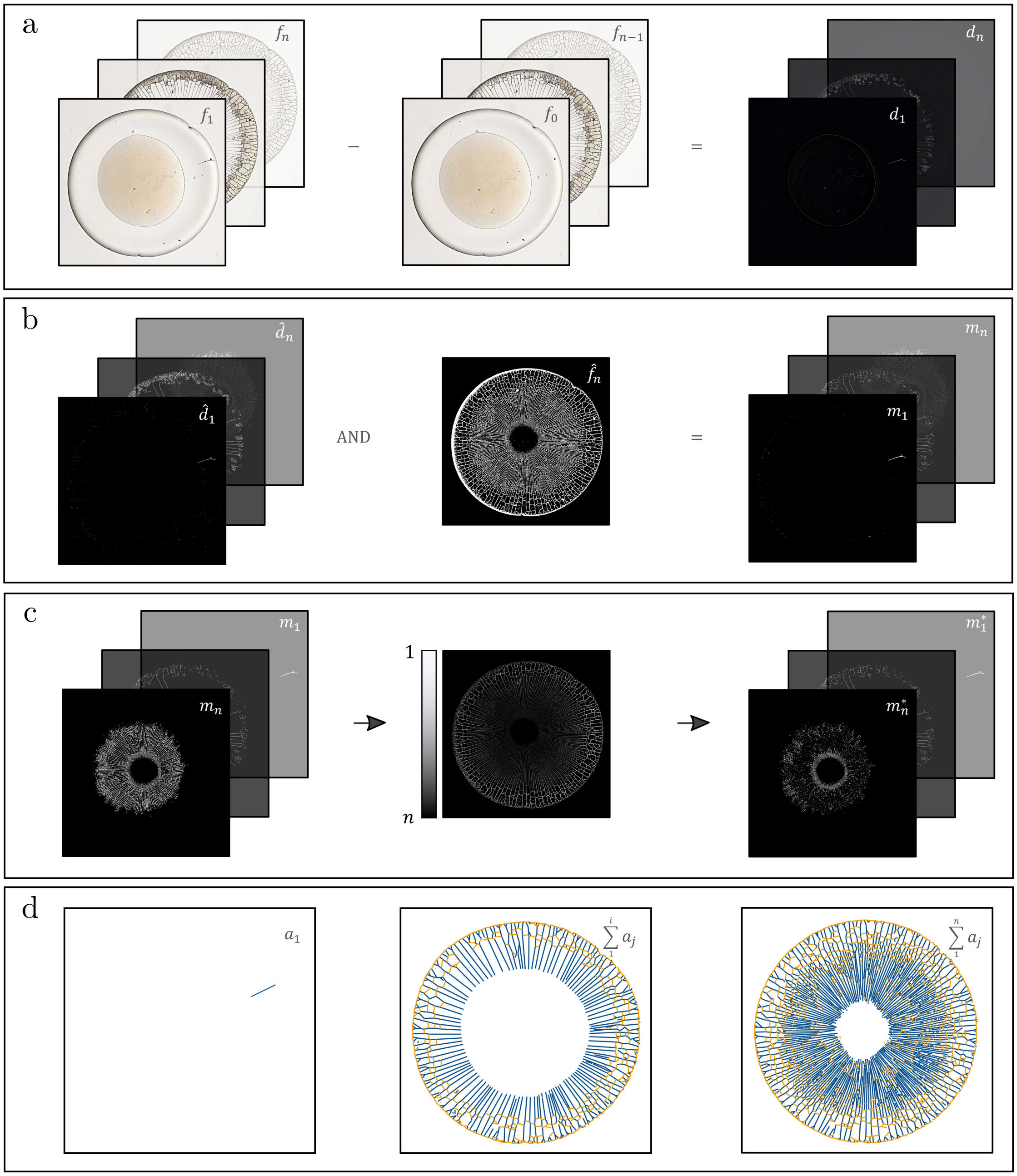

Fig. S3. Time-resolved crack identification. (a) Original images $(f_i)_{1,\ldots,n}$ saved at a one second interval and difference images $(d_i)_{1,\ldots,n}$. (b) Thresholded difference images $(\hat{d}_i)_{1,\ldots,n}$ and thresholded final image $\hat{f}_n$. Images $(m_i)_{1,\ldots,n}$ are obtained by a pixel-wise AND operation. (c) Reversed stack $(m_i)_{n\ldots1}$ and flattened image where stack location is encoded in the intensity data. Each pixel location in the reconstructed stack $(m^*_i)_{n\ldots1}$ has a value of 1 a maximum of one time. (d) Analyzed cracks found in images 1 to i. Radial cracks are shown in blue and orthoradial cracks are shown in orange.

S4. Deposit delamination

Deposit delamination has been proposed as a mechanism for the saturation of the crack density *(54)*. The argument is that if the deposit starts to separate from the substrate, additional drying stresses are released *via* delamination instead of additional crack formation *(54)*. To test whether delamination is the cause of the saturation of the crack density in our experiments, we image the dried deposit shown in Fig. S4a using interference microscopy with monochromatic light of wavelength $\lambda = 532\,\mathrm{nm}$ *(20)*. Regions of the deposit that have delaminated from the substrate show constructive and destructive interference fringes, as shown in Fig. S4b. The fringes are caused by interference between light reflected at the top of the substrate and light reflected at the bottom of the deposit. A bright constructive interference fringe appears when the deposit has delaminated a distance $\lambda/4 = 133\,\mathrm{nm}$ above the substrate.

The delamination visible in Fig. S4 is very limited and only occurs for a fraction of segments towards the edge of the deposit. This indicates that most of the deposit either does not delaminate or lifts up less than $133\,\mathrm{nm}$, and suggests that delamination is not the primary mechanism for crack density saturation in our experiments.

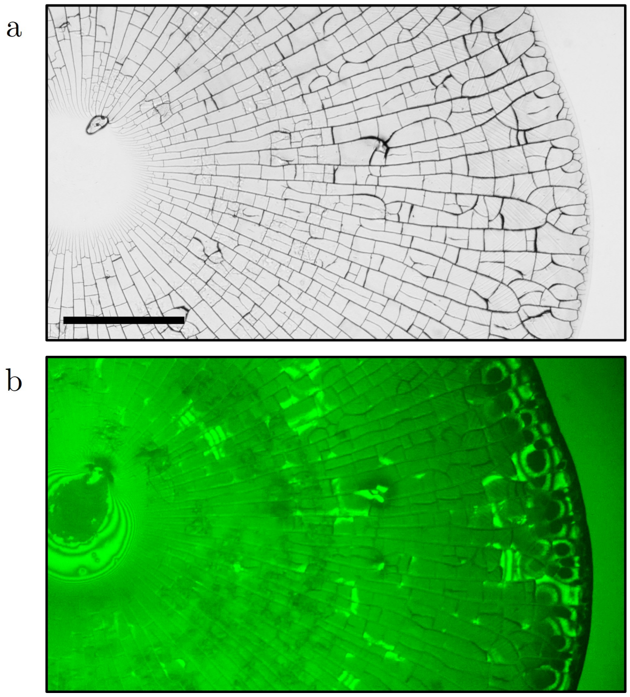

Fig. S4. Limited deposit delamination. (a) Transmission microscopy image of the final crack pattern for a drop with initial particle volume fraction $\phi_0 = 0.10$. The scale bar represents $200\,\mu\mathrm{m}$. (b) Interference microscopy image of the same drop. We use monochromatic light of wavelength $\lambda = 532\,\mathrm{nm}$. Brighter and darker fringes in cracked segments indicate that the deposit has delaminated and lifted from the substrate. Fringes are also visible in the center of the drop where the deposit thickness is of the order of λ.

Supplementary Movie Legends

Movie S1: Crack pattern formation for a drop with initial particle volume fraction $\phi_0 = 0.10$. Bottom-view video of the drop pictured in Fig. 1c, showing the formation of radial and orthoradial cracks. The video is played in real time and starts at $t = 150\,\mathrm{s}$ after deposition.

Movie S2: Crack pattern formation for a drop with initial particle volume fraction $\phi_0 = 0.05$. Bottom-view video of a section of the drop pictured in Fig. 3, showing the formation of radial and orthoradial cracks and the air invasion following orthoradial crack formation. The video is played in real time and starts at $t = 139\,\mathrm{s}$ after deposition.

Movie S3: Crack propagation and new crack formation. Bottom-view time-lapse at one second intervals of the section of the $\phi_0 = 0.05$ drop shown in Movie S2. Sections of radial cracks that have propagated or formed over the one second interval between two consecutive images are highlighted in red.
